# Supplementary material for: Mesencephalic Locomotor Region and Presynaptic Inhibition during Anticipatory Postural Adjustments in People with Parkinson’s Disease
Source: Brain Sci. 2024 Feb 15;14(2):178. doi: 10.3390/brainsci14020178 (PMC10887111; doi:10.3390/brainsci14020178)
Supplement: Supplementary file 1 [file brainsci-14-00178-s001.zip › brainsci-2854255-supplementary.pdf]

1. **Details about measuring the leg lifting task in an event-related functional magnetic resonance imaging (fMRI) to assess anticipatory postural adjustment (APA)**

Participants performed the task of lifting the right leg in a supine position inside the MRI scanner. Participants raised their leg from the hip, which required anticipatory downward pressure with the opposite leg to stabilize the pelvis prior to leg lifting. The movement of the leg was constrained by an adjustable brass bar placed 1 cm above the right foot to reduce the amount of head movement. Lying inside the scanner, participants were asked to follow the same stimuli and order as during the step initiation task (cross, black and white circles). Commands were presented on a mirror over the subjects' eyes, reflecting a screen 2.5 m away from them. As previously published,[4] the duration of each stimulus was randomized through Poisson probability distribution to improve the estimation of the hemodynamic response function (HRF) during brain volume acquisition.(Hagberg *et al.*, 2001) Duration of the first stimulus (cross – to relax) ranged 5.5-8.5 s (interstimulus interval). Duration of the prompt stimulus (first circle, black or white) ranged 1-3 s in steps of 0.5 s. The imperative stimulus to raise the leg (second circle, black or white) was presented for up to 5 s - the stimulus vanished at the onset of the leg lifting (identified by a pressure sensor placed in the support base). Participants performed 30 trials of the leg lifting task in the MRI scanner. A light-coupled trigger was used to synchronize the stimuli, vertical forces applied by the right foot, and the movement of the left foot with brain volume acquisition.(de Lima-Pardini *et al.*, 2017) Head motion was prevented by using pillows, pads and tapes around the head. In addition, velcro-connected bands were used to prevent trunk motion. See Figure 4 in our previously published article(de Lima-Pardini *et al.*, 2017) showing the experimental setup in the scanner. APA onset was defined as the time onset of a steep increase in vertical force (leg lifting task) determined as 2 standard deviations above the mean of the baseline force (average of the previous 500ms). Leg lift onset was defined as an abrupt drop in vertical force of the pressure sensor for the right moving leg. APA amplitude was defined as the maximum force value subtracted from the force value at APA onset. APA amplitude in the scanner was normalized by the average of the 10 frames preceding APA onset (baseline). APA amplitude is dimensionless. Individuals were excluded from the analyses if: a) they performed more than 30% of trials (10 trials) with multiple APAs inside the scanner, which might be associated with actual motor arrests; b) they did not lift the right leg in more than 50% of trials (15 trials), which it was impossible to detect the amplitude of the medio-lateral force.

REFERENCES

de Lima-Pardini AC, de Azevedo Neto RM, Coelho DB, Boffino CC, Shergill SS, de Oliveira Souza C, Brant R, Barbosa ER, Cardoso EF, Teixeira LA, Cohen RG, Horak FB & Amaro E, Jr. (2017). An fMRI-compatible force measurement system for the evaluation of the neural correlates of step initiation. *Scientific reports* **7**, 43088.

Hagberg GE, Zito G, Patria F & Sanes JN. (2001). Improved detection of event-related functional MRI signals using probability functions. *NeuroImage* **14**, 1193-1205.
